# Supplementary material for: Effectiveness of Educational Interventions on Adherence to Lifestyle Modifications Among Hypertensive Patients: An Integrative Review
Source: Int J Environ Res Public Health. 2020 Apr 7;17(7):2513. doi: 10.3390/ijerph17072513 (PMC7177836; doi:10.3390/ijerph17072513)
Supplement: Supplementary file 1 [file ijerph-17-02513-s001.pdf]

Table S1. Summary of included studies in chronological order

Authors	Year	Country	Participants	Sample size	Interventions	Outcome measures
Chiu & Wong [28]	2010	China – Hong Kong (High-income)	● From a family medicine clinic.<br>● Female 66.7%, Male 33.3%<br>● Age 53.87 ± 7.65 years.<br>● Attrition rate: 1.6%	C:32, I:31	An 8-week nurse-led programme.<br><br>C: One individual 45 minutes consultation in nurse clinic covered the contents of HTN management.<br>I: In addition to the individual consultation as control group, five to eight nurse-initiated phone calls were made to assess the patient's need and reinforce the self-management behaviours during the span of 8 weeks.	● Office blood pressure measurement.<br>● Self-developed adherence assessment scale.<br>● Satisfaction questionnaire.
Tobari et al. [29]	2010	Japan (High-income)	● From a community primary care center.<br>● Female 34.1%, Male 65.9%<br>● Age 61.75 ± 7.74 years.<br>● Attrition rate: 3%	C:64, I:64	A 24-week pharmacist-led programme.<br><br>C: A 20-minute pharmacist counseling of HTN management. Received a related educational leaflet.	● Office blood pressure measurement.<br>● Sodium reduction score.<br>● Level of daily exercise.

					I: Added on the interventions in control group, six 15-minute individual counseling was provided every month. The individual counseling established individual goals of lifestyle modifications.	
Hacihasanoglu & Gözüm [32]	2011	Turkey (Middle-income)	• From three public primary health care facilities.<br>• Female 51.7%, Male 48.3%<br>• Age 56.27 ± 8.27 years.<br>• Attrition rate: 7.6%	Three arms C:40, Ia:40, Ib:40	A 24-week nurse-led programme.<br><br>C: Six individual interviews to check blood pressure and weight without any educational intervention. Phone calls were made as reminder for next appointment.<br>Ia: Total six 30-minute monthly educations on medication adherence and general HTN management, 4 in clinic and 2 at home. Monthly nurse-initiated phone call to provide information about hypertension.	• Office blood pressure measurement.<br>• Medication adherence self-efficacy scale.<br>• Health-promoting lifestyle profile.

					Ib: Total six 45-minute monthly educations on medication adherence, further revised lifestyle modifications and general HTN management, 4 in clinic and 2 at home. Monthly nurse-initiated phone call to provide information about hypertension.	
Ribeiro et al. [33]	2011	Brazil (Middle-income)	• From a primary health care unit.<br>• All female<br>• Age 53.15 ± 5.57 years.<br>• Attrition rate: 9.6%	C:14, I:14	A 20-week team-based programme.<br><br>C: Five monthly group nutritional educations to address the dietary approaches on HTN.<br>I: With the same nutritional educations as control group, five home visits were provided in three months to address the needs of each family. The visits lasted 30-60 minutes.	• Office blood pressure measurement.<br>• Food frequency questionnaire.<br>• Consumption of oil, sugar and salt.

Authors	Year	Country	Participants	Sample size	Interventions	Outcome measures
Beune et al. [30]	2014	Netherlands (High-income)	● From four primary health care centres.<br>● Female 52.5%, Male 47.5%<br>● Age 53.93 ± 9.88 years.<br>● Attrition rate: 4.8%	C:68, I:71	A 20-week nurse-led programme.<br><br>C: Received standard HTN care and education in line with the recommendations in the local guidelines.<br>I: In addition to the interventions in control group, three 30-minute individual culturally appropriate counseling conducted by a nurse at week 2, 8 and 20 to address the HTN management.	● Office blood pressure measurement.<br>● A modified lifestyle adherence scale.<br>● Morisky medication adherence scale.<br>●
Katsarou et al. [26]	2014	Greece (High-income)	● From two university hospital-based outpatient clinics.<br>● Female 61.1%, Male 38.9%<br>● Age 65.43 ± 11.46 years.<br>● Attrition rate: 20%	C:20, I:16	A 6-week team-based programme.<br><br>C: An approximate 15 minutes standard care on medication and lifestyle modifications.<br>I: Three 90-minute group educations on stress management and Mediterranean diet principle were	● Office blood pressure measurement.<br>● Mediterranean diet score (Med score).<br>● International physical activity questionnaire (IPAQ-short form).

					provided by a psychologist and a nutritionist at week 2, 4 and 6.	
Ma et al. [34]	2014	China (Middle-income)	● From one hospital-based health centre and one community-based health centre.<br>● Female 50.8%, Male 49.2%<br>● Age 58.76 ± 11.03 years.<br>● Attrition rate: 11.6%	C:60, I:60	A 24-week nurse-led programme.<br><br>C: A standard health education on HTN management delivered by cardiologists or specialist nurses every 6 weeks. Related leaflets were provided.<br>I: Eight individualised theory-based counseling were delivered by trained nurses at home or health center. Each session lasted 30-40 minutes to assess the patient's performance and provide strategies to improve adherence on lifestyle modifications.	● Office blood pressure.<br>● Treatment adherence questionnaire of patients with hypertension. (TAQPH).
Friedberg et al. [31]	2015	United States (High-income)	● From the clinics under a medical group in two sites.<br>● Female 1.8%, Male 98.2%<br>● Age 65.89 ± 9.52 years.<br>● Attrition rate: 9%	Three arms C:180, Ia:177, Ib:176	A 24-week team-based programme.<br><br>C: A standard information about HTN management at enrollment.	● Office blood pressure.<br>● Physical activity recall.<br>● Willett food frequency questionnaire.

					Ia: Standard information about HTN management at enrollment, and six individualised monthly phone call. Each phone call lasted 15 minutes to provide standard HTN management information and other healthful behaviors.<br>Ib: Standard information about HTN management at enrollment, and six individualised monthly phone call by counselors with postgraduate degree in psychology or social work. Each phone call lasted 30 minutes and was based on the progress of a theory to address an individual's lifestyle modifications on HTN management.	● Dietary approaches to stop hypertension score (DASH score).
Paula et al. [35]	2015	Brazil (Middle-income)	● From the outpatient department in a hospital.<br>● Female 55%, Male 45%<br>● Age 62.1 ± 8.3years.	C:20, I:20	A 4-week team-based programme.<br>C: Individual counselling on diabetic dietary recommendations.	● Office blood pressure.<br>● Pedometer.<br>● Dietary records.

			• Attrition rate: 0%		I: Individual counselling on anti-hypertensive dietary recommendations and physical activity. Phone call or message reminders were made twice weekly to reinforce the adherence to the protocol.	
Cornelio et al. [36]	2016	Brazil (Middle-income)	• From an outpatient department in hospital and a healthcare center.<br>• All female<br>• Age 59.9 ± 10.1years.<br>• Attrition rate: 22.7%	C:49, I:43	A 12-week nurse-led programme.<br><br>C: Routine HTN management according to the health institutions. (No further information provided)<br>I: Two 60-90 minutes group workshops on salt limitation were provided every month. A cookbook and measuring spoon were provided. Phone call was made every 15 days after the workshop to enhance the effect of interventions.	• Dietary salt consumption.<br>• Psychosocial determinants of salt consumption.

Authors	Year	Country	Participants	Sample size	Interventions	Outcome measures
Arani et al. [37]	2017	Iran (Middle-income)	● Non-admitted patients from a hospital.<br>● Female 76.2%, Male 23.8%<br>● Age 54.06 ± 7.93 years.<br>● Attrition rate: 0%	C:40, I:40	A 3-week nurse-led programme.<br><br>C: Routine HTN management. (No further information provided)<br>I: Regards of routine HTN management, five theory-based group education on lifestyle modifications in HTN were provided by an expert nurse. Each session lasted 60 minutes and was held twice a week.	● Lifestyle questionnaire.
Wan et al. [38]	2018	China (Middle-income)	● From 5 neurology departments in 3 hospitals.<br>● Female 34.8%, Male 65.2%<br>● Age 35-86 years, 58.8% aged 60 or above.<br>● Attrition rate: 9%	C:78, I:80	A 12-week nurse-led programme.<br><br>C: Standard health education with handout provided. Nurse-initiated telephone follow-up after 1 week and 4 weeks of discharge.<br>I: Followed the similar protocol as control group, but the content was theory-based. An individual theory-driven health education	● Office blood pressure.<br>● Modified health-promoting lifestyle profile.

					lasted 20-30 minutes on lifestyle modifications. A theory-based handbook was provided. Two theory-driven phone calls lasted 15 minutes were conducted at week 1 and 4 after discharge to deliver health education. Twelve weekly message reminders were sent to reinforce the lifestyle modifications.	
Ayodapo & Olukokun [39]	2019	Nigeria (Middle-income)	• From an outpatient department in a hospital.<br>• Female 50.6%, Male 49.4%<br>• Age 60.9 ± 10.01 years.<br>• Attrition rate: no information.	C:161, I:161	A 12-week team-based programme.<br><br>C: No information provided.<br>I: One individual counseling on lifestyle modifications was provided and lasted for 30-45 minutes. Phone calls and message reminders were used to reinforce the adherence on lifestyle modifications biweekly for 12 weeks.	• Office blood pressure.<br>• Lifestyle behavior questionnaire.

HTN = hypertension; C = Control group; I = Intervention group
